# Multidimensional Representation Dynamics for Abstract Visual Objects in Encoded Tangram Paradigms

**DOI:** 10.3390/brainsci15090941

**Published:** 2025-08-28

**Authors:** Yongxiang Lian, Shihao Pan, Li Shi

**Affiliations:** Department of Automation, Tsinghua University, Beijing 100084, China; lianyx20@mails.tsinghua.edu.cn (Y.L.);

**Keywords:** electroencephalography, visual cognition, tangram paradigm, cognitive-associative encoding, multidimensional representation

## Abstract

Background: The human visual system is capable of processing large quantities of visual objects with varying levels of abstraction. The brain also exhibits hierarchical integration and learning capabilities that combine various attributes of visual objects (e.g., color, shape, local features, and categories) into coherent representations. However, prevailing theories in visual neuroscience employ simple stimuli or natural images with uncontrolled feature correlations, which constrains the systematic investigation of multidimensional representation dynamics. Methods: In this study, we aimed to bridge this methodological gap by developing a novel large tangram paradigm in visual cognition research and proposing cognitive-associative encoding as a mathematical basis. Critical representation dimensions—including animacy, abstraction level, and local feature density—were computed across a public dataset of over 900 tangrams, enabling the construction of a hierarchical model of visual representation. Results: Neural responses to 85 representative images were recorded using Electroencephalography (*n* = 24), and subsequent behavioral analyses and neural decoding revealed that distinct representational dimensions are independently encoded and dynamically expressed at different stages of cognitive processing. Furthermore, representational similarity analysis and temporal generalization analysis indicated that higher-order cognitive processes, such as “change of mind,” reflect the selective activation or suppression of local feature processing. Conclusions: These findings demonstrate that tangram stimuli, structured through cognitive-associative encoding, provide a generalizable computational framework for investigating the dynamic stages of human visual object cognition.

## 1. Introduction

The human brain is capable of extracting multiple representations from visual objects with varying levels of abstraction, including color, contours, categories, and contextual associations, all within ∼200 ms [1,2,3,4,5,6]. The isolated temporal and spatial dynamics of these visual representations have been investigated extensively using neuroimaging techniques such as Magnetoencephalography (MEG), Electroencephalography (EEG), and functional Magnetic Resonance Imaging (fMRI). Previous studies have predominantly employed either simplified stimuli (e.g., color patches and geometric shapes) or natural images (e.g., faces, animals, and tools) depending on the targeted representation [7,8,9]. While such stimuli facilitate an analysis of specific effects, they inadequately capture the brain’s capacity to holistically learn and integrate multiple representations during object recognition. Simple images lack rich semantic information, and in actual experiments, natural images are difficult to quantify and design beyond a few representational dimensions (such as category, shape, and color), while it is also challenging to consider multiple feature dimensions simultaneously within feasible dataset sizes [10]. Therefore, conventional experimental datasets often fail to incorporate sufficient representation diversity, reflecting a lack of breadth that prevents the concurrent quantification of multiple representational dimensions. Representation depth can also be limited when participants possess substantial prior knowledge about common experimental stimuli, which complicates the design of controlled paradigms that track cognitive transitions from unfamiliarity to familiarity. Tangrams provide a unique methodological strength in visual cognition research [11,12,13]. The geometrically primitive composition of these structures preserves semantically meaningful information that aligns with human object cognition and enables both precise quantification and systematic parameter design, a dual advantage that is rarely offered by conventional stimuli. As such, critical representations that prove challenging for concurrent operations with conventional datasets (i.e., shape, category, local feature density, prototypical similarity, and continuous abstraction level) become computationally tractable with tangram datasets. As a result, the investigation of representation dynamics in well-encoded tangram sample sets offers unprecedented opportunities for exploring how the visual system coordinates and learns multiple representations during visual cognition processes.

Notable trends to use two or more highly correlated representation dimensions, acquired from existing studies, were considered when designing the visual stimulus dataset [14]. These studies demonstrated that samples combining two representation dimensions, such as the cortical co-distribution of aspect ratios and categories, shape-semantic associations, and links between visual perception and conceptual cognition (e.g., human vs. primate faces, geometric shapes and categories, or images and concepts) [15,16,17,18,19] provide critical insights into visual representation mechanisms, specifically highlighting how multiple representational dimensions are integrated in the brain to support coherent cognitive representations. However, deeper investigations into visual object cognition require experimental paradigms that are capable of simultaneously manipulating multiple representation dimensions while capturing dynamic cognitive processes, a challenge that remains unmet by current stimulus datasets. For example, most of the representation dimensions contained in natural images are too flexible to quantify effectively, such as the categorization of local features, the varying contribution of local features to object recognition, and the overall difficulty of image recognition, as these factors are highly context-dependent and lack standardized metrics [15]. In contrast, the tangram, an ancient and widely recognized cognitive training tool, inherently incorporates rich representation dimensions and clear structures [20]. The inclusion of seven basic geometric components allows for the straightforward quantification of low-level visual features while the combinatorial configuration engages higher-order cognitive processes. Empirical studies have identified multiple representation dimensions that are operationally present in tangram tasks, including color, shape, category, abstraction level, local features, and configural novelty [11,20,21]. Notably, the unique capacity of tangrams to parametrically characterize continuous abstraction gradients (continuous distribution of abstraction parameters) during cognitive processing [22] directly addresses a key factor in multidimensional representation integration. Furthermore, the structural organization of tangram stimuli exhibits intrinsic compatibility with established theories of hierarchical visual cognition and perceptual grouping principles [23]. This congruence enables a direct mapping between the mathematical structure of tangrams and the computational framework underlying visual object cognition.

**Figure 1:**
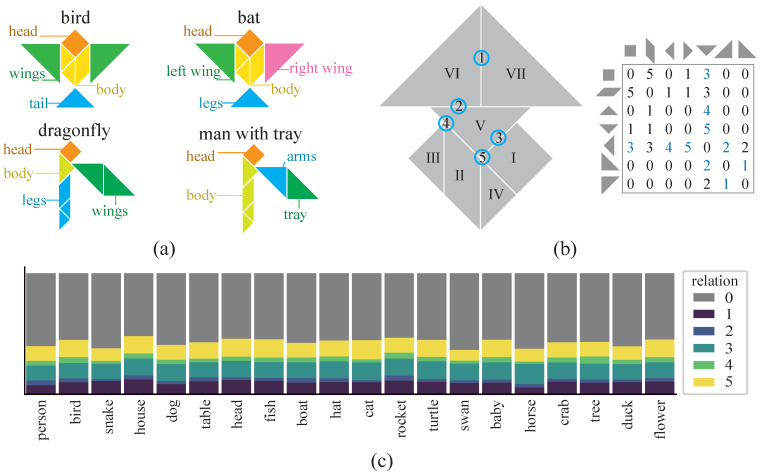
The tangram dataset and cognitive-associative encoding. (**a**) An example of tangram annotations, each of which consists of at least 10 global and segmentation annotations. (**b**) Cognitive association encoding, in which Roman numerals are used to label the seven basic component shapes in the tangram image on the left. The numbered labels correspond to 6 types of binary relationships, ranging from 0 to 5: no overlap, complete line overlap, partial line overlap, line containment, point-line contact, and point overlap. The numbering order reflects the cognitive closeness of each binary relationship. The corresponding 7 × 7 weighted adjacency matrix is shown on the right, where 0 indicates no connection between two basic shapes. The blue font in the matrix corresponds to the blue circular areas on the tangram. (**c**) The distribution of cognitive association encoding across different annotations. The top 20 most frequent global annotation words were selected and the lengths of bars of different colors represent the proportions of these five binary relationships. The color coding corresponds to the binary relationships shown in panel (**b**).

In this study, we employed a large-scale published tangram dataset named KILOGRAM for use as the experimental stimuli (Figure 1a), comprising over 900 well-annotated tangram images. We also established a mathematical framework with graph theory for hierarchical representations by first building on perceptual organization principles in computational vision and visual object cognition [24]. Specifically, guided by perceptual organization principles [25,26], we defined and validated cognitive-associative encoding through computational task evaluations and EEG experiments. This robust mathematical framework enabled systematic investigations of multiple critical representation dimensions. Beyond basic categories, we quantified abstraction levels, local feature density, and various types of local features such as head-like and wing-like shapes. Multivariate decoding successfully dissociated distinct representation dimensions across visual objects while capturing dynamic cognitive processes modulated by familiarity levels. This tangram paradigm demonstrated unprecedented capacity for co-manipulating multiple representation dimensions within dynamically evolving cognitive contexts. As such, this novel approach provides the potential to investigate the neural mechanisms underlying visual abstraction hierarchies, particularly the temporal coordination between low-level feature processing and high-level conceptual integration.

## 2. Materials and Methods

### 2.1. Cognitive-Associative Encoding

First, to interrogate multidimensional visual representations with precise experimental control, we formalize a cognitive-associative encoding that offers a graph-theoretic description of each tangram’s local structure and high-level semantics. This encoding operationalizes the target dimensions (e.g., animacy, abstraction, local-feature density) and provides the model features used throughout subsequent analyses. A multidimensional representation structure was first established in the tangram dataset by developing a cognitive-associative encoding to describe visual objects. We then defined visual objects as collections of local features and structural relationships, based on hierarchical visual processing and perceptual organization principles [25,27]. Specifically, in the tangram dataset, local features were naturally described by the composition of seven geometric primitives (one square, one parallelogram, and five triangles). These structural relationships could then be defined as six types of binary relationships between primitives, categorized according to human cognitive conventions (Figure 1b). Each tangram image was then quantified as a weighted connectivity graph and mapped into a 7 × 7 matrix (Figure 1b). As a result, this cognitive-associative encoding exhibits both scale and rotation invariance. The encoding also retains controlled degrees of image freedom (e.g., limb rotation around pivot points), while constraining critical local connections and global structures, thereby accommodating the requirements of visual object cognition research.

**Figure 2:**
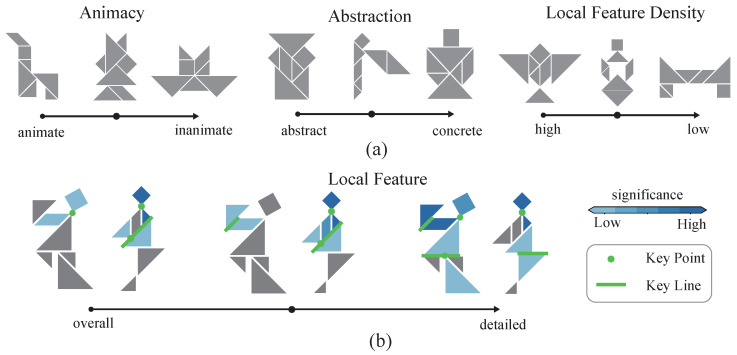
Examples of multidimensional representations in the tangram dataset. (**a**) Sample tangrams corresponding to continuous variations of different representation dimensions. From left to right: animacy, abstraction level, and local feature density. The arrows below each representation indicate the direction of continuous change. (**b**) Changes in local features across different levels of abstraction. Note that semantic abstraction gradually decreases from left to right, while the focus on local details increases. The saliency of each local component and key binary connections are also annotated in the figure.

The effectiveness of the proposed cognitive-associative encoding step was validated by conducting unsupervised and supervised evaluations (See Appendix A for details). A distance metric was first defined between two weighted adjacency matrices, and a multidimensional scaling (MDS) dimensionality reduction [28] was then performed to examine the separability of animacy categories in the encoding space. Following established dataset evaluation tasks [12], we employed an annotation-matching step in which each trial required selecting the most semantically congruent tangram image from 10 candidates (Figure 1c). Previous studies have performed similar tasks using pixel encoding along with large-scale deep learning models [12,29,30]. In contrast, a support vector machine (SVM) classifier was then used to analyze the classification accuracy of cognitive-associative encoding in our work. The resulting performance was then compared with published work [12], specifically pixel-based encoding baselines and human behavioral responses. This cognitive-associative encoding was also used to quantify local feature characteristics in the tangrams. We first assessed the local feature density (LFD) of each tangram, defined as the relative contribution of local features to cognitive discriminability, using systematic manipulation of matrix granularity (Figure 2). Classification efficiency for both local and global tasks was then calculated by modulating the spatial scale of a weighted adjacency matrix. Decision tree architectures [31] were implemented in the complete dataset, including more than 900 tangrams, to model categorical discrimination across target dimensions of interest (see the Appendix B). Gini index optimization was then used to identify critical local connections and prototypical classification patterns, subsequently deriving categorical decision rules for each target dimension.

### 2.2. Visual Stimulus Dataset

**Figure 3:**
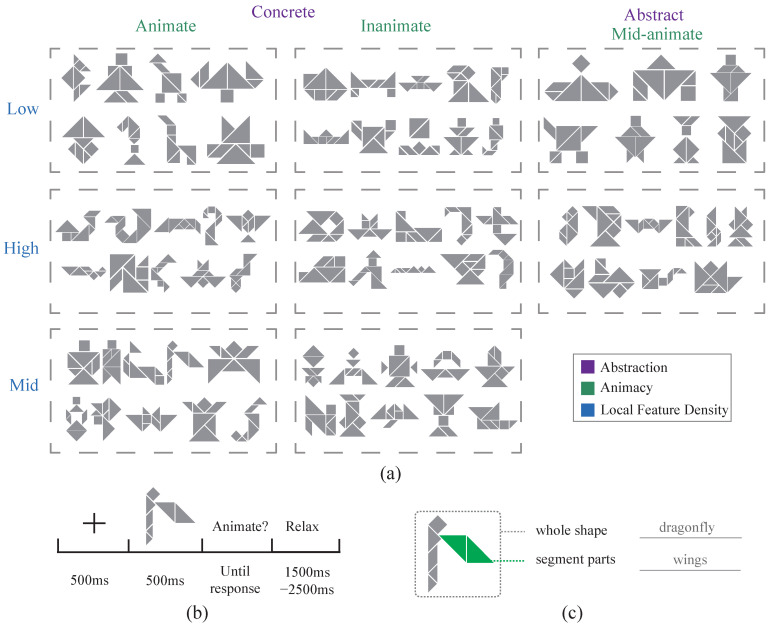
Dataset and experimental design. (**a**) Experimental stimuli, consisting of 85 images categorized along 3 different dimensions: animacy (animate, inanimate), abstraction level (abstract, concrete), and local feature density (high, medium, low). (**b**) The EEG experimental paradigm, including all 85 images presented in a randomized order, following a classic inter-stimulus interval paradigm. Participants were asked to determine whether the presented stimulus depicted an animal by pressing a key. (**c**) Two-step tangram annotation tasks conducted immediately after the EEG experiment. Participants first provided a global annotation for each tangram, followed by a detailed segmentation annotation.

Encoding quantification and stimulus selection were conducted with a publicly available tangram dataset named KILOGRAM [12] containing over 900 tangrams, each of which was labeled with at least 10 segmentation annotations. A cognitive-associative encoding framework was then implemented to calculate the distributional characteristics of core representation dimensions, including animacy, abstraction level, and local feature density. An experimental stimulus set comprising 85 images was curated through the selection of representation distributions and supplemented using a natural image control group, constituting the final EEG experimental dataset (Figure 3a). Natural images were selected from online sources where visual objects were easily segmentable, and the image categories were balanced across the dataset. Notably, the proposed technique facilitates a flexible adaptation of dataset filtering criteria according to specific research objectives. It also provides foundational mechanisms for generating novel tangram configurations through controlled parametric adjustments of the weighted adjacency matrix.

### 2.3. Subjects and Experimental Procedures

This study was approved for program review by the Medical Committee of the Science and Technology Ethics Committee at Tsinghua University (ID THU01-20240023) and informed consent was obtained from all participants. A total of 26 people (10 females; 16 males; ages 18–31; Mean = 24.9; SD = 2.7) participated in the experiment. The subjects were all right-handed and exhibited normal or corrected normal vision. EEG data from two of the subjects were excluded from some of the analyses due to excessive noise. The final data from 24 subjects were used for EEG analysis (9 females; 15 males; ages 18–31; Mean = 24.6; SD = 2.5). The experiment was conducted in a dimly lit, acoustically and electrically shielded room. Experimental samples were displayed on a 24-inch LCD monitor (resolution of 1920 × 1080, refresh rate of 60 Hz) located 65 cm from the participants. Visual stimuli were displayed centrally on a uniform gray background corresponding to a visual angle of ∼3°. The subjects first familiarized themselves with the stimulus information and the task using five practice images from the same encoded tangram dataset. In the formal experiment, participants made a total of 420 trials and judgments for 85 stimulus images (Figure 3b). Each image was presented 5 times across the experiment. All 85 images were shown once in randomized order before the next repetition cycle began. To ensure focus, the task was divided into 14 blocks with short breaks between blocks. In each trial, individual stimuli were presented for 500 ms after a center cross directed viewer attention. Participants judged stimulus animacy using button presses (with the left/right response balanced), and the response window was unrestricted. The inter-trial interval between the end of the key press and the subsequent stimulus presentation was randomized over a range of 1.5–2.5 s. Each block task typically lasted no more than 3 min, with the entire EEG experiment lasting ∼40 min. Immediately following the EEG experiment, participants performed corresponding detailed annotation tasks using 40 representative stimulus images from the experiment (Figure 3c), including both general annotation and segmentation steps. The total annotation time was ∼40 min.

### 2.4. Signal Acquisition and Preprocessing

**Figure A1:**
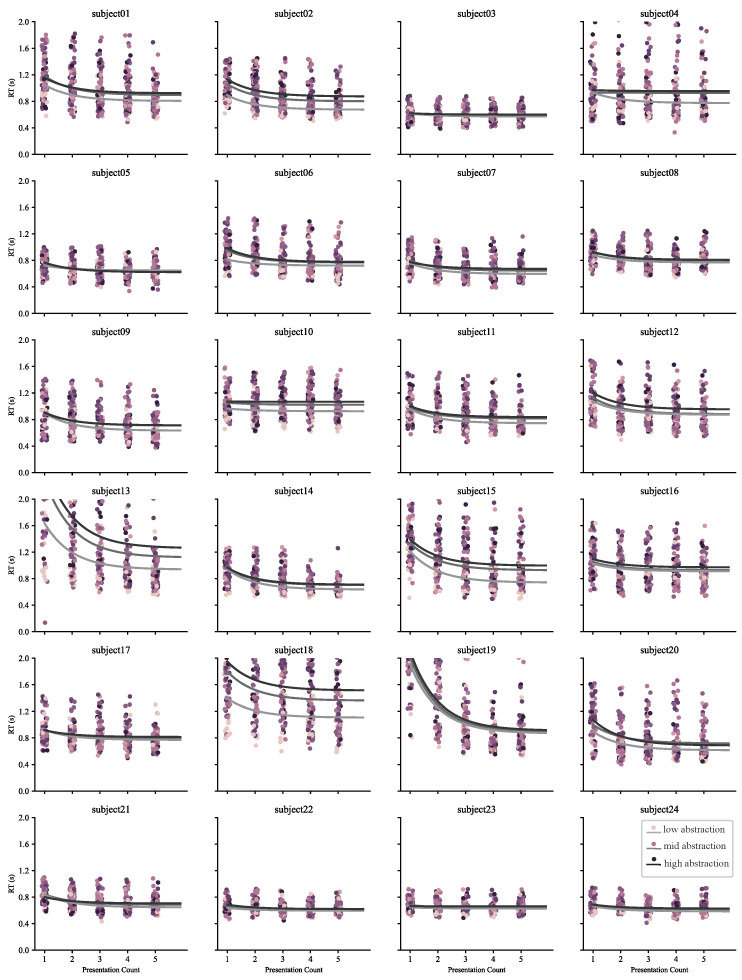
Distribution of response times for 24 subjects without normalization.

We collected EEG and behavioral data and applied standardized preprocessing to obtain artifact-clean, stimulus-locked epochs. These steps establish reliable univariate effects and produce the common data matrix for all downstream multivariate analyses. Continuous EEG data were acquired using an eego mylab system (EE-225; ANT Neuro, Hengelo, The Netherlands) equipped with a 64-channel gel Ag/AgCl electrode cap (CA-208; ANT Neuro) at a sampling rate of 500 Hz, integrated with synchronized markers generated by PsychoPy [32]. Electrode placement followed the international 10-10 system, with CPz used as a reference and AFz providing the ground. Electrode impedance was maintained below 5 kΩ throughout the experiment. After acquisition, EEG data from 61 channels (excluding EOG, M1, and M2) were preprocessed and analyzed offline using MNE-Python [33]. EEG data preprocessing involves several key steps used to ensure the accuracy and reliability of the resulting analysis. First, the samples were re-referenced to an average value and a bandpass filter from 0.1 to 40 Hz was applied to eliminate unwanted frequencies. The time period from −500 ms to 1000 ms was then segmented, relative to the appearance of the cross symbol, and baseline corrections were made using an average amplitude from −400 to −100 ms. Time periods with amplitudes exceeding ±80 μV on any channel except EOG were removed following standard procedures [10]). Independent component analysis (ICA) was applied using an automated detection algorithm to remove components related to eye and muscle artifacts [33]. For each participant, the original 420 epochs were reduced to a mean of 283.5 epochs (max = 341, min = 220, SD = 35.2). Since response time (RT) distributions varied considerably between subjects (Figure A1), RT analyses were conducted on all 420 trials per participant. Trials with excessively fast (<0.2 s) or slow (>5 s) responses were excluded. Trials with multiple key presses or premature responses were also removed. The same subset of trials was used for both behavioral and EEG analyses. Normalized reaction times were used for the analysis of multi-subject inter-group effects [34].

### 2.5. Decoding Analysis

A multivariate pattern analysis (MVPA) pipeline [35,36] was implemented for the preprocessed EEG voltage data. This analysis quantifies the temporal dynamics and interactions among dimensions beyond mean-level effects, linking representational features to neural separability. The input data to MVPA consisted of preprocessed epochs spanning 0–1 s following visual stimulus onset. This pipeline incorporated a regularized linear discriminant analysis (LDA) classifier with trial cross-validation included. A within-subject decoding step was then followed by group-level statistical integration. The analysis window spanned the entire stimulus presentation epoch used to investigate object representation dynamics. The temporal characteristics of multidimensional representation structures were also examined by comparing neural responses across distinct representation dimensions, including animacy, abstraction level, local feature density, and specific local features using classifier training and testing procedures. Subsequent decoding analysis focused on the primary dimensions, specifically animacy, abstraction level, and local feature density. Each dimension was then partitioned into high/medium/low tertiles, with classifier training incorporating covariate control for non-target dimensions. For condition assignment, epochs were partitioned according to stimulus properties, including participant-annotated categories, annotated abstraction levels, and local feature contributions computed via cognitive-associative encoding. Subsequent comparative analysis contrasted the decoding performance between computationally derived features (produced by cognitive-associative encoding) and human-annotated labels. Finally, image-level representation patterns were investigated using a stimulus-specific decoding strategy across all 85 images. The mean pairwise classification accuracy was calculated with a 50% chance-level baseline for all representation dimensional analyses [35]. Dynamic interactions between multidimensional representations were characterized by employing a temporal generalization analysis [37,38,39], in which the stimulus familiarity level (number of presentations) served as a critical contrast condition. Classifiers trained on all time points from high-familiarity trials were tested across all temporal samples in low-familiarity trials, with reciprocal cross-testing performed in reverse. The resultant temporal generalization matrices were then averaged across validation folds [40] and cross-validation was implemented for image-grouped classification contrasts to prevent data leakage between training/test sets [2,35]. All analyses utilized regularized LDA classifiers, with decoding accuracy averaged across cross-validation iterations.

### 2.6. Representational Similarity Analysis

**Figure 4:**
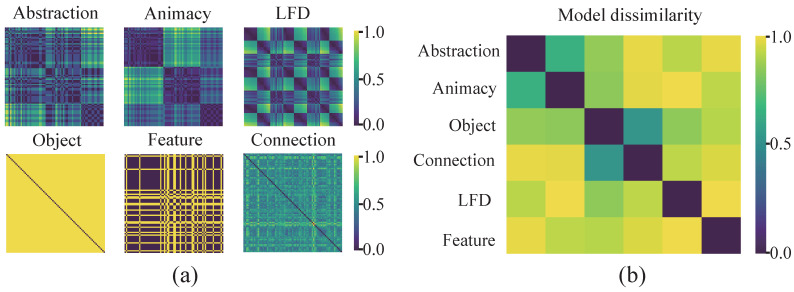

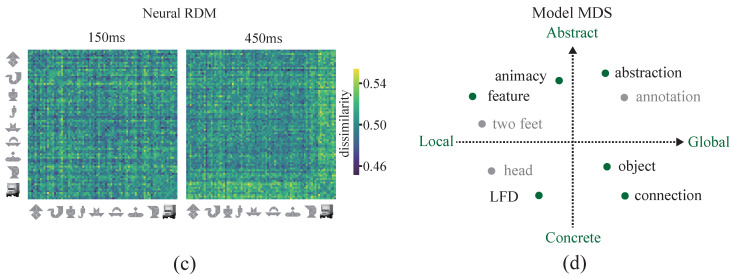
Models corresponding to multidimensional representations in RSA. (**a**) Representational dissimilarity matrices (RDMs) corresponding to different dimensions. The semantic models include abstraction level, animacy, and object categories, while the feature models include local feature density, local features, and connections. (**b**) Distance matrices for RDMs in each dimension, with distances calculated as a 1-correlation. (**c**) Neural signal RDMs at different time points. Each point in the 85 × 85 matrix represents the dissimilarity (decoding accuracy) between a pair of images. The 85 images were arranged into nine groups in the order shown in the figure. (**d**) Dimensionality reduction of representational distances using classical multidimensional scaling (MDS), in which model similarities are projected into a two-dimensional space. All representation dimensions exhibit a structured distribution along the axes of the image structure and semantic abstraction. In addition to the six candidate models, gray-colored dimensions are included for reference.

The representational structure within the tangram dataset was investigated using a representational similarity analysis (RSA) framework [41,42] for comparison of representation models. RSA complements decoding by testing correspondence at the level of representational structure, integrating evidence across methods and validating the proposed hierarchical organization. Image-level decoding outcomes were organized into 85 × 85 neural representation dissimilarity matrices (RDMs) (Figure 4a), in which each element quantified the mean cross-validated decoding accuracy between image pairs, with higher accuracy indicating greater neural dissimilarity. Subject-specific neural RDMs were then constructed for each time point (Figure 4c) and divided into six separate candidate models representing animacy, abstraction level, local feature density, object level, feature level, and connections of tangrams (see Appendix C for details). These models were then jointly visualized through MDS-based dimensionality reduction (Figure 4b,d), revealing two primary axes in the representational space: image-level structural variations and semantic abstraction gradients (See Appendix C for details). The unique contributions of each model to the neural dissimilarity were quantified by implementing a time-resolved GLM technique [36,43]. Vectorized lower-triangular neural RDM elements were then regressed against candidate model predictors for each time point and subject, yielding model-specific beta coefficients across subjects and temporal samples. The included group-level analyses focused on averaged beta estimates across participants. In each temporal window, a stimulus was embedded into the 2D space using t-SNE [28] applied to a mean neural RDM. This nonlinear projection preserved relative distances in the high-dimensional neural representation space, enabling visualization of dynamic clustering patterns aligned with semantic and structural dimensions.

## 3. Results

### 3.1. Cognitive-Associative Encoding Describes the Distributional Structure of Multidimensional Representations

**Figure 5:**
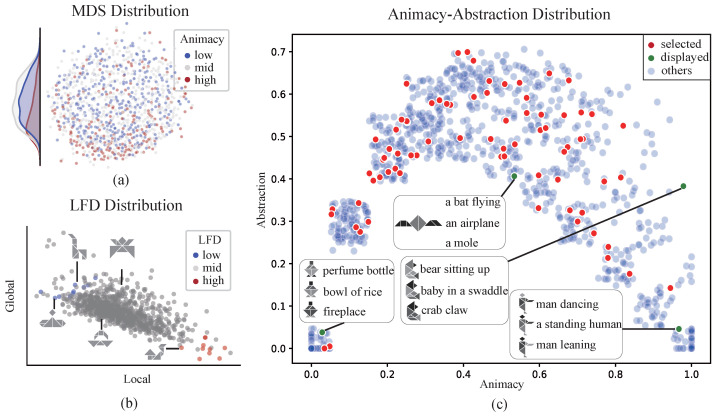
Distributions of multidimensional representations for the tangram dataset. (**a**) A visualization of dimensionality reduction for the semantic distribution in cognitive-associative encoding. Multidimensional scaling (MD) was used to embed cognitive-associative encoding into a two-dimensional space, with different animacy groups (i.e., low, medium, and high) specifically highlighted. The left panel shows the distribution of the three animacy groups along the vertical axis. (**b**) The distribution of local and global semantic contributions. Classification accuracy was calculated using weighted adjacency matrices at different scales, representing the contributions of local and global information. The final selection included three groups of local feature densities (i.e., low, medium, and high), excluding extreme values. Representative tangram examples are shown in the figure. (**c**) The distribution of animacy and abstraction level. The red dots indicate representative tangrams selected after filtering, while the green dots denote tangrams with representative annotations and displayed segmentations. Some overlapping data points were slightly shifted to enhance the visualization, without affecting the overall distribution.

The cognitive-associative encoding process was found to inherently exhibit semantic separability. By employing a distance defined on the weighted adjacency matrix, the MDS results revealed that tangrams with higher animacy tended to cluster toward the lower end of one dimension (t[987] = −6.75, *p* < 0.001) (Figure 5a). This suggests that the animacy dimension exhibits statistically significant separability within the cognitive-associative encoding, and therefore, cognitive-associative encoding naturally implies a description of animacy. Furthermore, a significant negative correlation was observed between local and global information (r = −0.644, *p* < 0.001), which aligned with both definitions and intuitive expectations (Figure 5b). Specifically, in the context of visual object cognition tasks, this indicates that the greater the contribution of local information, the smaller the contribution of global information, which corresponds to the intuitive expectation of a trade-off between detailed feature processing and holistic representation. Once the tangrams were quantified using a weighted adjacency matrix, calculation of the local information magnitude within the overall semantic context (i.e., local feature density) became straightforward. In representative cases, tangrams with a high proportion of local information exhibited prominent local features (Figure 2a), such as distinct heads, necks, mouths, and limbs. We further calculated two representation dimensions (animacy and abstraction level) for tangrams in the dataset and examined their distribution along these dimensions (Figure 5c). The resulting distribution followed an inverted-U shape, indicating that both very high and very low animacy levels were associated with higher recognizability, corresponding to lower levels of abstraction. Representative tangrams and their annotations also demonstrated that higher abstraction was accompanied by more diverse labeling. For example, the same tangram might be annotated as a bear, a baby, or a crab claw.

A matching task (see Materials and Methods) was also conducted to determine whether the cognitive-associative encoding contained sufficient semantic information to describe the tangrams.The results demonstrated that, compared with pixel-based encoding [12,29,30], cognitive-associative encoding enables much simpler models to achieve higher accuracy (Table 1). Notably, in the uncolored task (see Materials and Methods), cognitive association encoding performance reached human-level accuracy.

### 3.2. Behavioral Effects of Representation Dimensions

**Figure 6:**
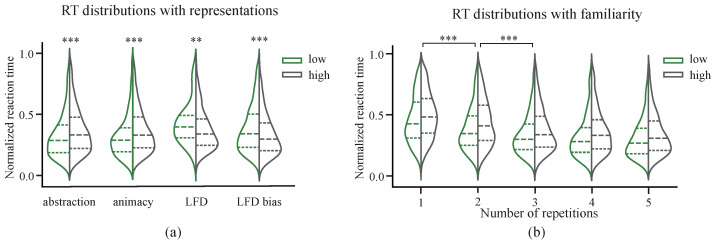
Normalized reaction times. (**a**) The distribution of normalized reaction times (RTs) for varying representation dimensions. From left to right: abstraction level, animacy, local feature density, and deviation of local feature density from the center. The dashed lines indicate the mean and the first quartile deviations from the mean. (**b**) The distribution of normalized RTs across stimulus repetitions. Significant differences in reaction times were observed only during the first three encounters with an image (FDR-corrected). As participants became more familiar with the stimuli, no significant differences in normalized RTs were observed in the 3rd, 4th, and 5th repetitions (** *p* < 0.01, *** *p* < 0.001).

Significant statistical variations were observed in participant response times across various representational dimensions (Figure 6a). As such, a nonparametric Wilcoxon test was primarily employed for statistical analysis. Results suggested that as image abstraction levels increased, average participant response times increased significantly (*p* < 0.001), likely representing the direct impact of increased recognition difficulty. Participants also recognized animal stimuli significantly faster than non-animal stimuli (*p* < 0.001), which may have been a result of more direct cues (e.g., head) available for animal identification. In addition, recognizing non-animal images required the exclusion of a larger variety of alternatives. Tangrams with prominent local features (higher LFD) were also recognized more rapidly (*p* = 0.007), which is consistent with previous reports in human visual object recognition [44]. Notably, the effects of the local deviation index, which describes deviations in the local feature density relative to a central value, were even more pronounced (*p* < 0.001). Higher values of this index indicate a predominance of either local or global information, suggesting that when one form of information is sufficiently dominant, it facilitates the recognition of abstract objects. Conversely, lower values reflect a coupling of local and global information and appear to impose an additional cognitive burden on the recognition process. Furthermore, a negative correlation was observed between the number of stimulus presentations and participant response times (r = −0.28, *p* < 0.001), which exhibited a significant decrease from the first to the second presentation and then stabilized from the third presentation onward (Figure 6b). Similar repetition effects have been reported in visual recognition literature [45], describing the cognitive processes associated with varying levels of familiarity. Both low- and high-abstraction images demonstrated reduced response times with repeated presentations. However, given the same presentation conditions, tangrams with higher abstraction consistently elicited longer response times than those with lower abstraction (Figure 6b).

### 3.3. Decoding the Dynamics of Representation Dimensions

**Figure 7:**
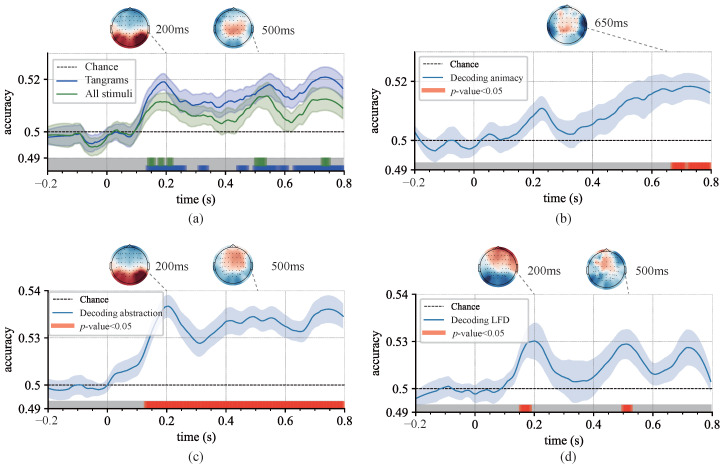
Mean decoding accuracy across representation dimensions. (**a**) Mean pairwise decoding accuracy for all stimulus images. The results are separated into two groups, one including natural images and the other containing only tangram images. The top row shows the topographic distributions of decoder channel weights at representative time points. The color bars beneath the curves indicate time intervals in which decoding accuracy is significantly above the baseline (*p* < 0.05). The shaded area around the curve represents the SEM. (**b**) Dissimilarity matrices for the neural RDMs at different time points. Each point in the 85 × 85 matrix represents the dissimilarity (1 − correlation) between a pair of images. These 85 images were arranged into nine groups in the order shown in the figure. (**c**) Decoding accuracy for animacy. The classifier distinguished between animate and inanimate categories. (**d**) Mean pairwise decoding accuracy for local-to-global feature ratios. The results are grouped into three levels based on local feature density (i.e., low, medium, and high, excluding extreme values).

Above-chance decoding results were observed across all classification tasks. Specifically, the decoding of category and abstraction level dimensions began 100 ms after stimulus onset for the set of 85 images that included natural shapes (Figure 7). In contrast, the decoding of local feature density began slightly later at 130 ms (Figure 7d). This temporal difference may be attributed to the decodability of low-level visual features present at the image level, as previous studies have reported onset times for various category decodings between 80 ms and 100 ms [46]. The onset observed in this study was marginally delayed, possibly due to relatively uniform low-level features (e.g., colors, connections, curvatures) in the tangrams. The decoding peak for animacy was observed at 500 ms, while other representation dimensions exhibited peaks at both 200 ms and 500 ms. Although these results are largely consistent with previous findings, there is a slight delay compared to earlier reports [6]. Scalp topography analyses of channel decoding weights also revealed that electrodes over the occipital region exhibited greater weights at 200 ms, while at 500 ms, the central-parietal electrodes became more prominent. These patterns align with established visual processing, corresponding respectively to early visual information processing and later stages of feature integration and decision-making [10,47].

**Figure 8:**
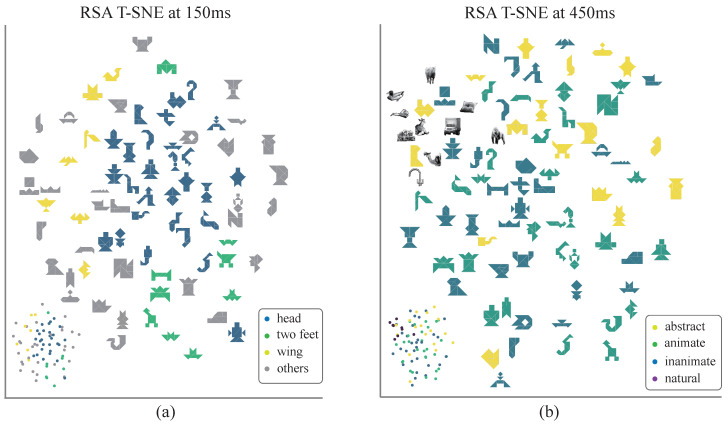
The representational RSA structure. (**a**) The distributions of various local features (e.g., head, body, legs, etc.) at 150 ms. Stimulus images were embedded in a two-dimensional space, reflecting their pairwise distances at various time points. The colored dots in the lower-left corner of each plot indicate the same distribution. (**b**) Distance distributions for various abstraction levels and categories (i.e., abstraction level, animate, and inanimate) at 450 ms. Stimuli were embedded in a two-dimensional space to reflect their pairwise relationships at this time point.

Similar temporal patterns were observed when decoding was performed exclusively on the subset of 75 tangram images without natural shapes included, though the overall significance of decoding was reduced (Figure 7a). Decoding outcomes for the abstraction grouping were particularly robust, underscoring the advantage of tangrams in studies of visual abstraction. The prolonged temporal window and delayed peaks observed in the decoding results were also consistent with longer response time of the subjects, highlighting the potential for more detailed temporal analyses and suggesting the cognitive processing of abstract visual objects may involve additional stages. A two-dimensional embedding map was also constructed of the stimulus images, based on neural representational dissimilarity matrices (RDMs), to qualitatively and intuitively explore the dynamics of individual representational dimensions. Embedding results were investigated at two time points, 150 ms and 450 ms, which corresponded to peaks in neural representation decoding strengths for the local feature and animacy models, respectively (Figure 8). In these embedding maps, the spatial distances between images reflected the average neural representation dissimilarity across participants. Early in processing, local feature separability was pronounced, while at later stages, higher-level dimensions (e.g., animacy) dominated the neural representations.

### 3.4. Cognitive Processes and the Effects of Multidimensional Representation Associations

**Figure 9:**
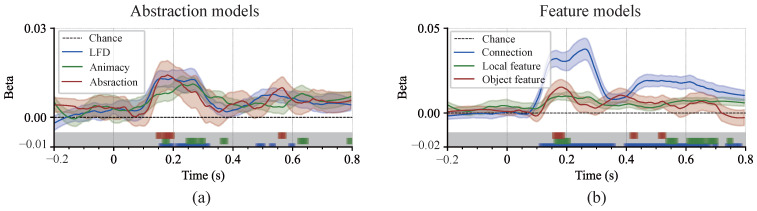
The results of an RSA model regression test. (**a**) The group of image features. Each participant’s neural RDM was regressed onto a linear combination of six candidate models, including three semantic models (abstraction, animal-likeness, and object category) and three feature models (local feature density, local features, and connections). The solid lines represent the estimated beta values for each model. The color bars beneath the curves indicate time intervals in which the beta values were significantly different from zero (*p* < 0.05). All values have been FDR-corrected. The shaded areas represent the standard error across participants. (**b**) The semantic abstraction group.

The representational structure of all stimulus images was investigated within a representational similarity analysis (RSA) framework [6]. We performed GLM analysis on two model groups: image structure and semantic abstraction (see Materials and Methods). Average beta estimates of these candidate models were then analyzed (Figure 9). This visualizes the dynamic time-domain contribution of a set of different models to the cognitive process. Initial neural responses were primarily captured by connectivity and abstraction models. Specifically, low-level connectivity features were represented early in the signal (∼160 ms), while the abstraction model also successfully accounted for variations in the early phase. Following these steps, representations related to local features and corresponding local feature densities emerged. Finally, semantic categorization based on animacy was observed (∼190 ms) (Figure 9a). These results quantitatively delineated the temporal contributions of various representational dimensions in abstract visual objects and underscored the hierarchical organization inherent in abstract visual cognition [48].

**Figure 10:**
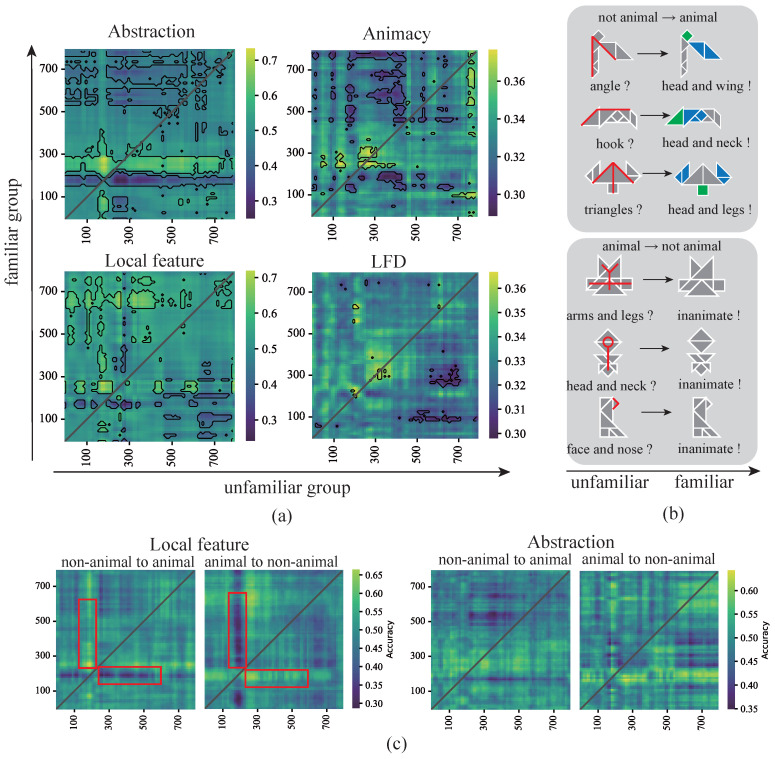
Time generalization effects for varying levels of familiarity. (**a**) Time generalization matrices for different representational dimensions across familiarity levels. The dimensions include abstraction (abstract vs. concrete), animacy (animate vs. inanimate), local features, and local feature density (low, medium, and high). The x-axis represents the unfamiliar group, and the y-axis denotes the familiar group. Clusters in which classification accuracy was significantly above or below chance are outlined. The above-chance generalization (yellow) above the diagonal indicates that processing under familiar conditions was slower than under unfamiliar conditions. (**b**) Specific examples of changes in decision-making under different familiarity levels. The top cases demonstrate that participants changed their responses to classify an image as an animal (corresponding to local feature activation). The bottom cases indicate that participants changed their responses to non-animal (corresponding to local feature suppression). Color blocks and lines reflect the local features of the subject’s feedback. Examples were selected based on participant responses and annotation data. (**c**) A comparison of time generalization effects between two types of decision change processes. The left panel shows the temporal generalization effects for local feature activation versus suppression, while the right panel compares temporal generalization effects for abstraction levels. The time generalization results (especially within the red box) exhibit a certain degree of symmetry along both sides of the diagonal.

A time generalization analysis was also conducted for each representation dimension across presentations groups. Trials were then divided into unfamiliar and familiar outcomes, based on different distributions of response time. Generalization in abstraction-level decoding was predominantly driven by the familiar group. Specifically, a 200–300 ms time window in the familiar group generalized to a 150–200 ms window in the unfamiliar group (Figure 10a). This finding indicated that under unfamiliar conditions, participants exhibited an earlier abstraction-related process, which became progressively suppressed as familiarity increased, possibly due to the diminishing influence of abstraction caused by repeated exposure. A similar pattern was observed for animacy representations. The 200–300 ms window in the familiar group also generalized to the unfamiliar group, although the effect was concentrated at ∼300 ms in the unfamiliar group, which is consistent with previous findings on categorical representations [9]. In the case of decoding measures pertaining to image structure (i.e., local feature density and local features), the 200–300 ms window in the familiar group similarly generalized to the 150–200 ms window in the unfamiliar group (Figure 10a). A more pronounced suppression was also observed for early generalization from the familiar group to points past 500 ms in the unfamiliar group. This suggests that under familiar conditions, participants exhibited an earlier local feature-related process, while the corresponding process is attenuated in unfamiliar conditions. This time generalization analysis further revealed consistent below-chance generalization between early and late responses, in agreement with previous visual object categorization studies [6] and attributable to stimulus offset, adaptation, or inhibitory signals [2].

Two representative cognitive processes associated with changes in participant response were identified in the repeated presentations of tangrams (Figure 10b). In the first process, participants did not initially classify the tangram as an animal during the first two presentations but changed their determination in the subsequent three presentations. Based on the trials of all subjects (see Appendix D for details), 11.5% of tangram images exhibited “change-of-mind”, corresponding to 8.625 out of 75 images. Participant feedback following the EEG experiment indicated that these participants focused on specific local features after repeated exposure (reported by 19 out of 24 participants), an effect referred to in this study as the cognitive activation process for local features. Conversely, in the second process, participants reversed their initial decision, no longer classifying the tangram as an animal after multiple presentations, thereby abandoning the local features recognized in the early trials. We termed this occurrence the cognitive inhibition process for local features. Trials corresponding to these two altered decision events were extracted for time generalization analysis, revealing symmetric patterns (Figure 10c). In the generalization analysis of local features, the unfamiliar group in the inhibition process dominated generalization, in contrast to the activation process. This is likely because, in the inhibition process, participants confirmed local features during earlier presentations but subsequently reversed their decisions. A similar pattern was observed in the generalization results for abstraction, in which the unfamiliar group in the inhibition process continued to dominate generalization.

## 4. Discussion

The current study leveraged tangram stimuli with cognitive-associative encoding to expand the representation dimensions of human visual object recognition, while capturing underlying cognitive processes. This dual approach addresses a methodological gap in traditional stimulus materials (e.g., simple stimuli and natural images) used to study the ways in which humans integrate and learn multidimensional representations [16,17,18]. Validated cognitive-associative encoding was used to define and quantify the key representation dimensions of tangram stimuli, including animacy, abstraction level, local feature density, and local features. These dimensions were embedded within a representational space spanning image structure and semantic abstraction and were successfully decoded above chance from EEG responses across 85 stimulus images. RSA model testing revealed that early neural responses primarily relied on low-level structural features, while later processing became increasingly associated with semantic category information and abstraction, which is consistent with hierarchical theories of visual cognition. Furthermore, trials in which participants changed their interpretation of a given tangram led to the identification of two distinct time-generalization patterns, one reflecting local feature activation and the other indicating local feature suppression, thereby capturing the bidirectional nature of cognitive shifts. These findings highlight the potential of the tangram paradigm for facilitating a deeper investigation into the ways in which humans integrate and learn multidimensional representations, offering novel insights into the temporal dynamics of abstract visual object recognition.

Behavioral statistical analysis also revealed differences in participant response times across groups defined by different representation dimensions. Notably, this effect within the abstraction level grouping was particularly prominent, highlighting the advantages of the tangram dataset in providing a quantifiable and continuous distribution of abstraction levels. In addition, local feature density, an important but typically difficult to quantify representation dimension in conventional datasets, exhibited coupling effects for behavioral analysis under the tangram paradigm. Both salient local features and global structural information facilitated faster cognitive processing, which supported a two-stage cognitive model [49]. Furthermore, response times gradually decreased and stabilized with repeated stimulus presentations, aligning with established findings in visual cognitive processing research [45]. Since the extensive prior knowledge of participants is inherently uncontrollable, existing studies based on simple geometric shapes and natural images often employ pretraining strategies to collect steady-state data [15,16,17], thereby failing to capture the cognitive process itself. In contrast, the proposed experimental paradigm effectively demonstrated an ability to track the cognitive processing dynamics of individual participants.

Decoding results for individual representation dimensions in the tangram stimuli revealed distinct temporal dynamics. Early decoding activation (100–150 ms) was primarily associated with low-level visual features, such as abstraction and local feature density [6]. In contrast, category-related representation dimensions linked to higher-level cognition (e.g., animacy) emerged later, at ∼400 ms. Notably, local feature density achieved peak decoding performance at ∼200 ms, while animacy representation reached its peak at ∼450 ms. This pattern is consistent with hierarchical cognitive theories, which suggest that higher-level categorical perception requires the accumulation of sufficient low-level representational information [44]. Interestingly, local feature density, which is associated with both low and high level features, displayed a bimodal decoding peak at approximately 200 ms and 500 ms, reflecting the temporal distinction between different levels of representational processing. Category-organized patterns were also observed at various time points using the RSA framework to qualitatively examine representation structures. At 150 ms, the embedded representational structure primarily represented local shape patterns, with prominent clusters corresponding to head-like, leg-like, and wing-like structures. By 450 ms, the structures exhibited a clear distinction between animate and inanimate objects, with natural images forming a well-segregated group. Such temporal representation transitions were commonly observed in neural responses within the ventral temporal cortex [8,43,50] and have been shown to align well with human categorization behavior [51,52]. This pattern may reflect the encoding of a continuous biological category gradient in the human brain [53]. The proposed paradigm also demonstrates alignment with existing research [18] on common representational structures while extending the scope of investigation toward additional critical dimensions, such as abstraction level and local feature density.

RSA regression and temporal generalization analysis provided rich detail describing dynamic relationships among multiple representation dimensions. As an extension of single representation dimension decoding results, the regression analysis of both image features and abstract semantic groups demonstrated early dominance by low-level models (i.e., local features and connectivity), which is consistent with previous research [6]. During two specific “changed mind” processes, the experimental paradigm successfully captured the dynamic aspects of cognitive processing. The temporal generalization analysis reflects these findings. In the transition from rejecting to affirming animacy, the familiar category dominated generalization, reflecting the activation of local features. Conversely, in the transition from affirming to rejecting animacy, local features were inhibited and the unfamiliar category governed generalization. The temporal order observed here highlights a predominantly bottom-up process: early phases are driven by local feature encoding, which serves as a prior to guide subsequent interpretation. Later reversals reflect feedback-based adjustments, where accumulated context or higher-level expectations reshape the contribution of these priors [15]. Unlike traditional steady-state experiments using conventional stimuli, the tangram paradigm effectively captures detailed dynamics of cognitive processes. This expansion of representation dimensions and the ability to track dynamic cognitive stages not only broadens the scope of research, it also provides new opportunities for a deeper understanding of visual object recognition.

Finally, the design of the experimental paradigm needs to be discussed in detail in terms of image selection and presentation. In recent years, experimental paradigms utilizing designed graphical stimuli similar to tangram have been developed to explore visual inference processes in greater detail [54]. These stimuli are similar to tangrams but with much weaker semantic information. Such paradigms offer a viable computational framework for investigating cognitive mechanisms (e.g., generative replay) and experimental designs, offering great potential for advancing research in higher-level visual processing. The rapid serial visual presentation (RSVP) paradigm has been increasingly adopted in recent visual mechanism studies to expand the number of trials [10]. However, applying this paradigm to the tangram task carries higher risks. Specifically, previous research has shown that for common visual stimuli, RSVP allows for more efficient processing without significant information loss [5]. However, tangram tasks pose greater challenges due to their abstract configurations and the increased demand for integrating local and global features. Compared to natural image stimuli, the decoding peak for tangrams exhibits a slight delay of approximately 50–200 ms, while the increase in response time is even more pronounced, suggesting that recognizing tangrams requires more extensive processing. The extremely brief presentation time for each individual image in RSVP does not allow participants to complete the full cognitive processes necessary for tangram recognition, including the extraction of local information, the processing of abstract semantic features, and the potential trade-off between local and global information. Thus, our paradigm did not use RSVP, which is efficient and popular but does not reflect enough information in tangram.

Tangram images exhibit higher consistency across samples, compared to conventional stimulus datasets, which makes decoding neural responses more challenging. As such, ineffective results that cannot be clearly separated are more common. This necessitates more refined experimental group designs and more effective data analysis methods, which were organized with cognitive-associative encoding in this work. In addition, while this study employed the commonly used 64-channel EEG, quantitative RSA modeling surpassed the level of representational structures typically obtainable from a standard EEG. Future work could integrate this experimental paradigm with EEG recordings involving more channels or methods that are more sensitive to spatial patterns, such as MEG, fMRI and the EEG source localization techniques [55]. Finally, the primary objective of this study was to demonstrate the potential of the proposed experimental paradigm. To this end, we collected a moderately sized dataset and employed analysis methods that primarily addressed the basic effects of multidimensional representations. A more detailed investigation into the specific mechanisms related to these representational dimensions will require richer datasets, more refined theoretical models, and thorough analytical validation.

## 5. Conclusions

The results presented in this study highlight the unique ability of the large-scale tangram dataset, structured through cognitive-associative encoding, to facilitate the simultaneous exploration of multiple representation dynamics and their underlying cognitive processes. This encoding strategy effectively isolated key representational dimensions—such as abstraction level, local feature density, and animacy—and demonstrated their independent and dynamic expression during cognitive processing. Notably, the temporal generalization analysis uncovered novel insights into the cognitive processes of decision-making, particularly in relation to how the brain adjusts or revises decisions over time. Beyond empirical characterization, the tangram paradigm provides theoretical leverage: its representational depth (fine-grained local parts and connectivity) and breadth (abstract categories and animacy) enable rigorous tests of competing accounts of hierarchical vision. These findings underscore the power of the tangram paradigm to capture the temporal dynamics and complexity of visual object cognition. As such, future research on human visual cognition would greatly benefit from adopting encodable stimulus datasets like tangrams, which offer a robust framework for investigating the integration and learning of multidimensional representations in the brain. By parametrically manipulating local-feature priors and task context within the encodable paradigms, EEG, MEG, and fMRI studies can capture increasingly detailed cognitive processes, thereby offering a pathway toward more refined theories of visual object recognition.

## Figures and Tables

**Table 1:** An accuracy comparison of tangram semantic matching tasks under different encodings. A 10-choice tangram annotation matching task [12], primarily comparing cognitive-associative coding and pixel coding. An SVM method provided results with different kernel functions (i.e., Linear, Polynomial, and Gaussian), while the Clip model provided both pretraining and post-fine-tuning results. The tangrams were divided into two groups: colored and black. The annotations were also divided into two groups: whole labeling and part labeling. A total of four comparison cases were combined and the highlighted results are marked in red.

Condition	Cognitive-Associative Code			Pixel Code		Human
	SVM_line	SVM_poly	SVM_gauss	clip_pt	clip_ft	
Whole + Black	11.8	39.6	49.0	16.1	43.3	47.7
Parts + Black	12.4	41.3	50.3	16.4	45.3	49.1
Whole + Color	12.6	37.8	41.8	15.9	40.8	49.5
Parts + Color	12.3	40.3	43.6	15.0	45.4	63.0

**Figure A2:**
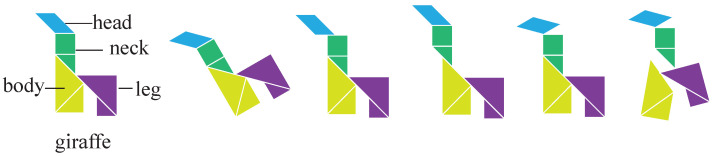
Demonstration of the advantageous properties of cognitive association encoding. In the figure, local parts of the original images were subjected to translation, rotation, and combinatorial transformations. Our encoding remained stable, exhibiting minimal variation in the encoding space, which is consistent with the stability of the underlying visual semantics.

**Figure A3:**
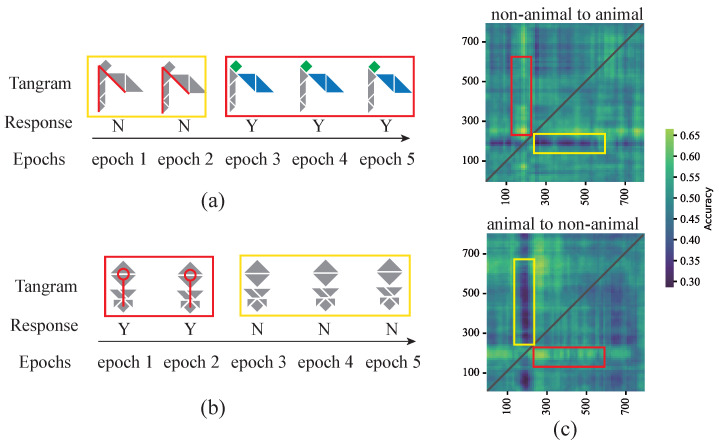
Examples of trials in which participants changed their mind. Yellow boxes indicate the suppression of local features, while red boxes indicate the activation of local features. (**a**) Change-of-mind from non-animal to animal. (**b**) Change-of-mind from animal to non-animal. (**c**) Temporal generalization analysis of both types of changes of mind.

## Data Availability

Annotations, stimulus sets, EEG data (raw and preprocessed), and the main analysis codes pertinent to this study are publicly accessible through the Open Science Framework (OSF) at https://osf.io/7qm35/ (accessed on 10 August 2025).

## Abbreviations

The following abbreviations are used in this manuscript:

EEG	Electroencephalography
fMRI	Functional Magnetic Resonance Imaging
MEG	Magnetoencephalography
EOG	Electrooculogram
RT	Response Time
LFD	Local Feature Density
MVPA	Multivariate Pattern Analysis
LDA	Linear Discriminant Analysis
RDM	Representational Dissimilarity Matrices
RSA	Representational Similarity Analysis

## Appendix B. Cognitive-Associative Coding

### Appendix B.1. Excellent Characteristics of Coding

Below, we elaborate on the advantageous properties of the cognitive association encoding. First, scale and rotation invariance: Our encoding is defined based on the binary relations between fundamental compositional units. Specifically, in the context of tangrams, these are the six types of relations between two basic shapes described in the Section 2, and the resulting encoding matrix can be regarded as a specialized adjacency matrix. Consequently, the cognitive association encoding inherits the properties of adjacency matrices, exhibiting invariance to scale and rotation.

Second, local stability: More importantly, the encoding demonstrates additional local stability. For instance, if two basic shapes have a point-contact relation, rotations around the contact point do not alter this relation until other parts overlap. The cognitive association encoding thus remains unchanged. Even in cases of substantial local variation, our encoding exhibits only minor changes. For example, the connection between the head and neck in certain stimuli remains stable under head rotations or swings, avoiding situations where semantic invariance leads to large changes in the encoding. In contrast, other representations, such as pixel-based encodings, cannot achieve this property.

Third, ordered encoding: Our encoding is not merely a categorical classification of basic connections. We assessed the cognitive relatedness of binary relations and defined an order among encoding types. From most to least related, the sequence is as follows: complete overlap of line segments, partial overlap, crossing, line-to-point contact, point contact, and complete separation. This ordering transforms the encoding from a discrete classification into a continuous space, facilitating the definition of distances and providing the foundation for subsequent dimensionality reduction and statistical analyses.

### Appendix B.2. Validation with MDS

We applied multidimensional scaling (MDS) to examine the low-dimensional structure of the cognitive association encodings of the stimuli. Each stimulus was represented by a cognitive association matrix and paired with its corresponding animacy rating.

Two types of distance metrics were defined: 1. Euclidean distance, computed directly from the cognitive association matrices, which captured the absolute structural differences between two tangram stimuli. 2. Encoding frequency distance, obtained by transforming each matrix into a distribution of connection types and then computing the dissimilarity between distributions, which reflected the similarity of structural composition profiles across stimuli.

For both distance metrics, we calculated the pairwise dissimilarity matrix and applied the MDS function from the scikit-learn library (sklearn.manifold.MDS), specifying two target dimensions (components = 2) under the metric MDS setting (metric = True). The resulting embeddings consistently revealed a clear separation along the animacy dimension across both distance definitions, suggesting that animacy is a salient organizing factor in the cognitive association space of the stimuli.

### Appendix B.3. Validation with SVM

We employed support vector machines (SVMs) to evaluate image–text matching performance in a 10-choice classification task. Each trial consisted of a tangram stimulus and 10 candidate textual labels, among which only one label was correct. The input pairs consisted of the cognitive association encoding of each tangram stimulus and its corresponding textual annotation, yielding more than 9000 image–text pairs in total. The dataset was randomly split into training and testing subsets with a 4:1 ratio.

The tangram stimuli were represented by their vectorized cognitive association encoding, while the textual annotations were encoded by the CLIP text encoder, producing semantic embedding vectors. The two representations were concatenated to form the feature input to the classifier. We trained SVM classifiers using three types of kernels: 1. Linear kernel (kernel = “linear”), 2. Polynomial kernel (kernel = “poly”), 3. Radial basis function (RBF) kernel (kernel = “rbf”). The implementation was based on the SVC function from the scikit-learn library (sklearn.svm.SVC), with the regularization parameter set to its default value (C = 1.0). Output: Model performance was evaluated by classification accuracy on the held-out test set, providing a measure of how well each kernel captured the relationship between cognitive association encodings and semantic labels.

### Appendix B.4. Validation with Decision Tree

We employed a decision tree model to extract prototypical local features and their corresponding cognitive association encodings, aiming to derive structural prototypes of visual objects. Participants’ annotations were categorized to identify frequently occurring local features (e.g., head, face, wing, handle, tail, foot). A non-overlapping tangram dataset was then constructed, ensuring that stimuli associated with different feature categories were separated. Each tangram stimulus was represented by its cognitive association encoding (vectorized), paired with its categorical label.

We implemented a C4.5 decision tree, which uses the information gain ratio as the splitting criterion. To control model complexity and enhance interpretability, the tree depth was explicitly constrained. In addition, a pruning strategy was applied based on the distribution of class labels in child nodes: the Gini index was computed, and nodes with impurity below a predefined threshold were not further expanded. This ensured that branches representing nearly pure class distributions were pruned, preventing overfitting.

The resulting tree 1. identified clusters of tangram stimuli sharing similar local features, 2. represented classification rule mapping cognitive association codes, local features to object categories, 3. provided a compact and interpretable structure after pruning. This approach revealed prototypical features for different categories (e.g., animal prototypes characterized by head/face/wing, tool prototypes by handle/foot), while the pruning strategy ensured that the extracted decision rules were both parsimonious and interpretable.

## Appendix C. Representation Dissimilarity Matrices

A neural representational dissimilarity matrix (neural RDM) was constructed for each participant at each time point, capturing the dissimilarities among all 85 stimuli. This neural RDM was modeled as a linear combination of six candidate models: low-level connectivity, abstraction level, animacy, local feature density, local features, and image-level models. These models were divided into two groups: image structure and semantic abstraction, corresponding to the two primary axes in the representational space.

The computation of each representational dissimilarity matrix (RDM) is equivalent to determining the distance between any two tangram stimuli. The low-level connectivity distance was defined as the Euclidean distance between two cognitive association encoding matrices. The abstraction level followed the definition in the experimental design, where for a given tangram image, it was quantified as the dispersion of its annotations across categories (e.g., the extent to which annotations were distributed among humans, animals, and non-animals), and the corresponding distance was defined as the difference in this dispersion. Animacy was defined consistently with the experimental design as the proportion of annotations labeling an image as an animal, with the distance given by the difference in these proportions. Local feature density was defined as described in the Section 2, and its distance measure was likewise the difference in this index between two stimuli. The distance based on local features was computed from categorical annotation vectors for each tangram, with categories consistent with the subsequent analyses, namely head, wing, foot, tail, and face. At the image level, each stimulus was treated as an independent object for RSA, resulting in an identity matrix as its RDM. The categorical partitioning of these RDMs was determined by their relative distances, which, according to the distribution shown along the two axes in MDS figure (Figure 4), were organized into image structure and semantic abstraction.

## Appendix D. Change-of-Mind Analysis

### Appendix D.1. Trials Screening

For each participant, we analyzed the preprocessed clean epochs by setting the k-th response as a boundary and separately computing the responses from the first k trials and from the subsequent trials (with k set to 2 or 3 to ensure stability of the results). We then calculated the difference between the mean responses of these two segments. A trial was defined as a “change-of-mind” under two conditions: (1) when the two segments showed completely different responses, i.e., one segment consisting entirely of “animal” judgments and the other entirely of “non-animal” judgments; or (2) when the two segments were almost completely different, allowing at most a single deviation in choice.
